# Syncytin, envelope protein of human endogenous retrovirus (HERV): no longer ‘fossil’ in human genome

**DOI:** 10.1080/19768354.2021.2019109

**Published:** 2022-01-12

**Authors:** Serpen Durnaoglu, Sun-Kyung Lee, Joohong Ahnn

**Affiliations:** a College of Natural Sciences, Hanyang University, Seoul, Republic of Korea; b Research Institute for Natural Sciences, College of Natural Sciences, Hanyang University, Seoul, Republic of Korea

**Keywords:** HERV, syncytin, COVID-19, placenta, cancer, neurodegenerative disease

## Abstract

Human endogenous retroviruses (HERVs) are ‘fossil viruses’ that resulted from stable integrations of exogenous retroviruses throughout evolution. HERVs are defective and do not produce infectious viral particles. However, some HERVs retain a limited coding capacity and produce retroviral transcripts and proteins, which function in human developmental process and various pathologies, including many cancers and neurological diseases. Recently, it has been reported that HERVs are differently expressed in COVID-19 disease caused by infection of severe acute respiratory syndrome coronavirus 2 (SARS-CoV-2). In this review, we discuss the molecular structure and function of HERV ENV proteins, particularly syncytins, and their conventional roles in human development and diseases, and potential involvement in COVID-19 regarding the newly reported mental symptoms. We also address COVID-19 vaccine-related infertility concerns arising from the similarity of syncytin with the spike protein of SARS-CoV-2, which have been proved invalid.

## Introduction

Human endogenous retroviruses (HERVs) represent 8% of the human genome (Lander et al. 2001). HERVs are derived from germline infections by exogenous retroviruses early in the evolution of primates (Barbulescu et al. 1999). A complete HERV is approximately 9.5 kb in length and consists of four essential viral genes; *gag, pro, pol,* and *env* flanked by two long-terminal repeats (LTRs) (Griffiths 2001; Khodosevich et al. 2002; Balada et al. 2009; Kim and Shin 2020). LTRs can act as promoters to drive HERV genes and contain regulatory sequences, binding transcription factors (Durnaoglu et al. 2021; Ito et al. 2017). A majority of HERVs in human genome are disrupted along the evolutionary path, mostly found in heterochromatin, and suppressed by epigenetic silencing at steady state (Rowe and Trono 2011). Expression of HERVs is often elevated in various physiological and pathological conditions (Dolei et al. 2019; Durnaoglu et al. 2021). In cancers, for example, a specific HERV *env* gene is activated and plays a crucial role in inducing carcinogenesis in certain malignant tumors, including breast cancer, pancreatic cancer, germ cell tumors, leukemia, and Kaposi's sarcoma (Alcazer et al. 2020; Gao et al. 2021). Syncytin-1 and 2, ENV proteins of HERV-W and HERV-FRD, respectively, are expressed in the placenta and involved in trophoblast formation (Dupressoir et al. 2012). In addition, syncytin-1 expression is elevated in neuropathological disorders such as schizophrenia (SZ) and bipolar disorder (BD) while positively correlated with C-reactive protein (CRP) levels, an inflammation indicator (Perron et al. 2008; Wang et al. 2018). CRP is an acute-phase serum protein, and its level rises when there is inflammation in the body (Du Clos and Mold 2004). Several factors increase the CRP level, such as infectious or non-infectious acute and chronic conditions, trauma, sleep disturbances, and periodontal diseases (Nehring et al. 2021).

Coronavirus disease 2019 (COVID-19) is a highly contagious viral illness caused by severe acute respiratory syndrome coronavirus 2 (SARS-CoV-2) (Lovato et al. 2020). Several symptoms include weakness, respiratory distress, muscle pain, sore throat, loss of taste and smell associated with the disease ranging from asymptomatic/mild symptoms to severe illness and even death. Although vaccination against SARS-CoV-2 helps protect from COVID-19, some people are hesitant to the vaccinations for a number of reasons, including a rumor that concerns a cross-activity of anti-SARS-CoV-2 spike protein syncytin-1 in the placenta. Also, some recent reports show the differentially expressed HERVs in COVID-19 patients, suggesting that HERVs may be involved in COVID-19 epidemiology (El-Shehawi et al. 2020; Kitsou et al. 2021; Marston et al. 2021). On the other hand, it has been reported that CRP level increases in serum from COVID-19 patients and positively correlates with disease severity (Chen et al. 2020; Luo et al. 2020). CRP is suggested as a factor contributing to the development of severe cases of COVID-19 by inducing the production of proinﬂammatory cytokines, apoptosis, and the inﬂammatory status (Mosquera-Sulbaran et al. 2021). In addition, CRP levels in COVID-19 patients with depression are higher than those without depression (Yuan et al. 2020; Lorkiewicz and Waszkiewicz 2021). Several meta-analyses have reported that patients with psychotic disorders have higher CRP levels than healthy individuals; however, the cause–effect relation is unclear (Miller et al. 2014; Osimo et al. 2021). There are also several cases of COVID-19 patients with elevated CRP levels, showing psychosis without a personal or family history (Ferrando et al. 2020; Smith et al. 2020; Łoś et al. 2021).

The review focuses on syncytins and CRP expression in mental disorders and the case reports with mental symptoms in COVID-19 patients. Furthermore, we summarize the scientific data, which confirms that there is no link between COVID-19 vaccines to problems related to pregnancy arising from the so-called similarity between syncytin and the spike protein of SARS-CoV-2 (Hillson et al. 2021; Kloc et al. 2021; Morris 2021; Rajak et al. 2021; Shimabukuro et al. 2021). Conversely, COVID-19 disease can hinder healthy fertility in both males and females (Jing et al. 2020; Shen et al. 2020; Cavalcante et al. 2021; Sharma et al. 2021; Vizheh et al. 2021). The insights from HERV activation in COVID-19 patients not only help understand the epidemiology of the pandemic with complicated symptoms, including mental disorders but also provide valuable information utilized in the development of therapeutic intervention of the viral disease.

## Syncytins

**Figure 1.**
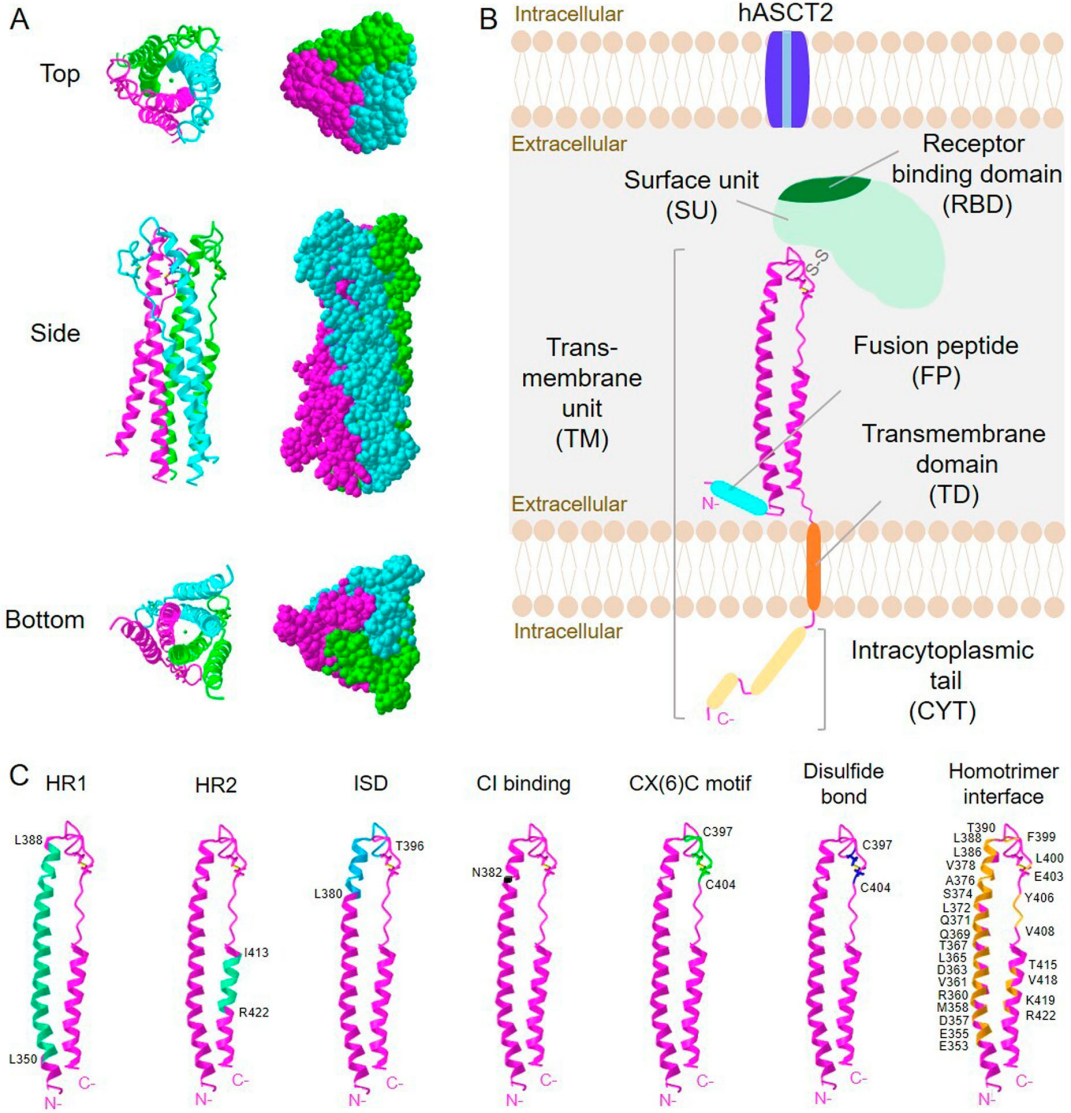
Structure of syncytin-1. (A) 3D crystal structure images of trimeric syncytin-1 are created with iCn3D structure viewer (Wang et al. 2020). PDB ID: 5HA6 (doi:10.2210/pdb5HA6/pdb). (B) Schematic representation of syncytin-1 monomer describing the surface unit (SU) and transmembrane (TM) units. Crystalized region shown in (A) is described in pink ribbon. The receptor binding site (RBD) is located in SU, and binds to the hASCT2 receptor to trigger membrane fusion. One disulfide bond links SU and TM. Fusion peptide (cyan blue), transmembrane domain (orange), and the intracytoplasmic tail (yellow) are indicated. (C) Functional sites in syncytin-1 are highlighted. Amino acid residues forming heptad repeats (HR1 and HR2), immunosuppressive domain (ISD), CI binding site, CX(6)C motif, a disulfide bond and homotrimer interface are labeled.

**Figure 2.**
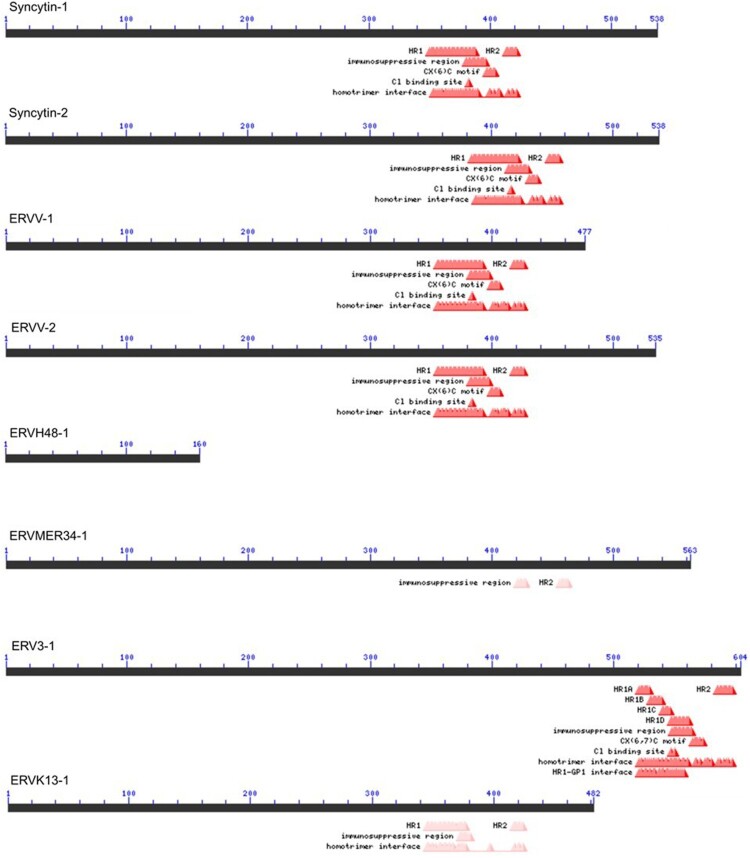
Syncytin family expressed in human trophoblast. Syncytin-1 and syncytin-2 are the *env* gene products of HERV-W and HERV-FRD, respectively. The coding genes are also known as ERVW-1 and ERVFRD-1, respectively. It has been shown that both syncytin-1 and -2 promote cell-cell fusion in placenta development. These two syncytins have all domains required for the fusion process; a fusion core made of heptad repeats (HR1 and HR2), CX(6)C motif, and homotrimerization interface. ERVV-1, ERVV-2, and ERV3-1 are little studied but are expressed in syncytiotrophoblast from early human embryos and in *in vitro* differentiated placental trophoblast (TB). ERVRH48-1 is known as suppressyn or SUPYN, expressed in unfused cytotrophoblast cells. SUPYN inhibits TB fusion *in vitro*, because it lacks all major domains and presumably acts as a competitor for other syncytins. ERVMER34-1 may inhibit promote fusion, too. ERVK13-1 encodes a long non-coding RNA transcript of unknown function. The domains are retrieved from NCBI's Conserved Domain Database (CDD) (Marchler-Bauer et al. 2017).

Some envelope glycoproteins encoded by *env* genes of human endogenous retroviruses (HERV) are expressed in the human placenta and play a critical role in early human development (Petropoulos et al. 2016; Yabe et al. 2016; Okae et al. 2018; West et al. 2019; Roberts et al. 2021). Those placental ENVs contribute to placental syncytial structures; Therefore, they are named syncytins (Figure 1). While the ENV protein products of ERVW1, ERVFRD-1, ERVV-1, ERVV-2, ERVH48-1, ERVMER34-1, ERV3-1, and ERVK13-1 are all detected in placental trophoblasts, most studied are HERV-W1 ENV product, the first identified syncytin-1, and ERVFRD-1 ENV product, syncytin-2 (Blond et al. 2000; Sha et al. 2000; Gong et al. 2005). These two syncytins share a very similar molecular structure that mediates effective membrane fusion (Figures 1 and 2).

### Syncytin-1

Syncytin-1 is a protein consisting of 538 amino acids, and HERV-W1 coding syncytin-1 is located at 7q21.2 of human chromosome 7. Syncytin-1 contains an N-terminal signal peptide of amino acids number 1 to 20 (aa 1-20), a surface unit (SU, aa 21-317), and a transmembrane unit (TM, aa 318-538). SU and TM contain functional domains for the protein’s physiological activities: receptor-binding domain (RBD), fusion peptide (FP), the fusion core N- and C-terminal heptad repeats (HR1 and HR2, respectively), immunosuppressive domain (ISD), transmembrane domain (TD), and intracytoplasmic tail (CYT) (Gimenez and Mallet 2008; Grandi and Tramontano 2018) (Figure 1).

Syncytin-1 is synthesized as a glycosylated gPr73 precursor that forms a homotrimeric structure (Figure 1). Syncytin-1 precursor is cleaved into two mature proteins: the gp50 SU and the gp24 TM (Gimenez and Mallet 2008). The cleavage takes place at the SU/TM junction, at a furin cleavage site of RNKR. Through a disulfide bond, SU and TM are covalently bonded and reach the cellular membrane. SU is in charge of recognizing and binding to cell receptors to initiate membrane fusion. The hydrophobic FP and fusion core consisting of HR1 and HR2 are presented in TM. HRs are also associated with homotrimerization. TM contains immunosuppressive domain (ISD), which is located inside HR2 and ends in the CYT. The CYT of the TM, FP, and fusion core consisted of HR1 and HR2 are all essential for the fusion process. ISD domain is suggested to confer syncytin-1 a critical role in fetal-maternal tolerance during pregnancy (Lokossou et al. 2014).

Syncytin-1 is highly fusogenic and actively involves trophoblast cell fusion and differentiation, thereby functioning in human placental morphogenesis critical for normal placental function (Blond et al. 2000; Frendo et al. 2003). Human placenta contains specialized trophoblast cells crucial for embryo implantation and placental development (Benirschke et al. 2012). At the early gestation stage, mononuclear cytotrophoblasts differentiate and fuse into a continuous layer of multinucleated syncytiotrophoblast. The syncytiotrophoblast layer is the site of various placental functions, including oxygen transport, nutrient exchange, immune tolerance, and the synthesis of hormones required for fetal growth and development. The fusogenic activity of syncytin-1 is activated upon interaction with the type D mammalian retrovirus receptor (Blond et al. 2000), referred to as hASCT-1/-2 (human sodium-dependent neutral amino acid transporter type 2) (Lavillette et al. 2002).

Syncytin-1 mRNA and protein levels are higher in the brain tissue from multiple sclerosis (MS) patients than in that from normal individuals. Syncytin-1 is specifically expressed in astrocytes, glial cells, and activated macrophages of MS lesions (Antony et al. 2004). *In vitro* expression of syncytin-1 induces the production of proinflammatory factors such as iNOS, IL-1β, and redox reactants in human fetal astrocytes (HFAs). Syncytin-1-expressing astrocytes are cytotoxic to oligodendrocytes, releasing redox reactants. In a mouse MS model, syncytin-1 mediates neuroinflammation and cell death of oligodendrocytes, which are prevented by antioxidant ferulic acid.

Also, syncytin-1 is implicated in several neural diseases. Syncytin-1 is highly expressed in activated immune cells in MS, but even higher during infections (Garcia-Montojo et al. 2020). Flow cytometry analysis of peripheral blood mononuclear cells (PBMC) from healthy donors, MS patients in relapse or remission, and patients with acute infections reveal that syncytin-1 is elevated in monocytes during MS relapses or acute infections. After stimulation with LPS, activated T and B lymphocytes and natural killer cells (NKs) increase expression levels of syncytin-1. The study suggests that syncytin-1 plays a critical role in the early stages of immune cell activation and may contribute to MS pathogenesis. In addition, overexpression of syncytin-1 in microglia induces the expression of inducible nitric oxide synthase (iNOS) and results in increased NO production, enhancing microglial migration (Xiao et al. 2017).

Syncytin-1 expression is elevated in the serum sample of patients with SZ and BD compared to healthy subjects (Perron et al. 2008; Perron et al. 2012). The level of CRP inflammation biomarker is correlated with syncytin-1 level. These results suggest that syncytin-1 plays a role in inflammatory regulation in neuropsychological diseases. It is reported that syncytin-1 triggers CRP activation via TLR3 signal cascade in glial cells (Wang et al. 2018). Overexpression of syncytin-1 increases CRP, TLR3, and IL-6 in both human microglia and astrocytes. Syncytin-1 induces expression of CRP and IL-6, but the induced expression is not observed in the lack of TLR3. Furthermore, syncytin-1 activates innate immunity by inducing the secretion of major proinflammatory cytokines such as IL-1β, IL-6, or TNF-α via CD14 /TLR4 in human monocytes (Rolland et al. 2006). Therefore, the tight association between the expression level of syncytin-1 and inflammatory events in glial cells may be involved in the etiology and pathology of psychotic diseases.

Syncytin-1 is also implicated in various types of cancers such as endometrial cancer, breast cancer, leukemia, urothelial cell carcinoma and human hepatocellular carcinoma, and enhances the cell proliferation, metastasis, and tumorigenicity (Yu et al. 2013; Sun et al. 2017; Chignola et al. 2019; Liu et al. 2018; Zhou et al. 2021). Syncytin-1 expression is associated with DNA methylation in the 5′LTR of the syncytin-1 gene in several cancers (Huang et al. 2014). In pancreatic cancer (Lu et al. 2015), decreased syncytin-1 expression is correlated with increased DNA methylation in the 5′ LTR region of syncytin-1 promoter, and syncytin-1 expression is reactivated when methylation levels in 5′LTR are reduced (Huang et al. 2014). Therefore, epigenetic changes affect the expression of syncytin-1, thereby participating in cancer development.

### Syncytin-2 in comparison with syncytin-1

Syncytin-2 is an envelope protein of the human endogenous retrovirus family HERV-FRD (Blaise et al. 2003; Grandi and Tramontano 2018). ERVFRD-1 encoding syncytin-2 is located at 6p24.1 of human chromosome 6. Syncytin-2 has all functional domains found in syncytin-1; SP (aa 1-15), SU (aa 16-350), and TM (aa 351-538), in which RBD, FP, HR1 HR2, ISD, TD, and CYT reside.

Syncytin-2 has been associated with one receptor, the major facilitator superfamily domain containing 2a (MFSD2a), a transporter for the essential omega-3 fatty acid (Nguyen et al. 2014). Like syncytin-1, syncytin-2 is also a fusogenic protein required for functional placental syncytia (Chang et al. 2004). While syncytin-1 is strongly expressed in villous syncytiotrophoblast, mainly in the basal membrane, but weakly in villous cytotrophoblasts, syncytin-2 is expressed in villous cytotrophoblasts (Roberts et al. 2021). Syncytin-2 is highly correlated with the degree of severity of preeclampsia (PE) (Vargas et al. 2011), a placental pathology characterized by maternal arterial hypertension, proteinuria, and organ dysfunction, in contrast to syncytin-1 in lesser degree (Bokslag et al. 2016). Both sequences of syncytin-1 and syncytin-2 harbor regions, which are highly similar to the conserved retroviral immunosuppressive domain (ISD) (Cianciolo et al. 1985; Kristensen and Christensen 2021). Syncytin-1 and syncytin-2 may be immunosuppressive via their association with placental exosomes (Tolosa et al. 2012; Lokossou et al. 2020). Peripheral blood mononuclear cells (PBMCs) cultured in the presence of syncytin-1-ISD then stimulated with lipopolysaccharides (LPS)/ phytohaemagglutinin (PHA) significantly reduces the production of the Th1 cytokines such as TNF-α and IFN-γ as well as the chemokine, CXCL1 (Park et al. 2020). Similarly, Jurkat cells were cultured with either syncytin-2-ISD region or trophoblast-derived exosomes and then stimulated with activating agents, phorbol 12-myristate 13-acetate (PMA)/ionomycin. Quantification analyses showed that induced Th1 cytokines, such as TNF-α, IFN-γ, and IL-2, were severely reduced. Thus, it is suggested that syncytin-1 and syncytin-2 contribute to exosome-mediated immunosuppression, reducing Th1 cytokine production in PBMCs.

## HERVs in human diseases

HERVs are implicated in various diseases, including some psychiatric conditions such as schizophrenia (SZ) and bipolar disorder (BD) and COVID-19, a viral infectious pandemic disease whose patients commonly suffer from psychological distress. Especially, the expression level of syncytin-1, the product of HERV-W1, is often changed in these illnesses and associated with those of inflammatory markers.

### Syncytin-1 in psychiatric diseases

Retroviral RNA of HERV-W family is detected in the cell-free cerebrospinal fluids (CSFs) of 10 of 35 individuals with recent-onset SZ or schizoaffective disorder but not in the CSFs of 22 individuals with noninflammatory neurological diseases or from 30 individuals with no neurological or psychiatric diseases (Karlsson et al. 2001). Increased transcription of HERV-W family RNA is found in the brain tissues from postmortem individuals with SZ compared to postmortem brain tissues from individuals without psychiatric diseases.

In a study done with 136 patients, 91 with BD, 45 with SZ, and 73 healthy controls, syncytin-1 transcription level is higher in patients with BD and SZ compared to healthy controls (Perron et al. 2012). In BD patients, the transcription level of syncytin-1 is also higher than in the patients with SZ. Syncytin-1 expression is detected in 23 of 49 patients with SZ, but only in 1 of 30 healthy controls (Perron et al. 2008). Syncytin-1 transcripts are detected in the plasma samples of 42 out of 118 patients with recent-onset SZ, and none is detected in 106 controls (Huang et al. 2011). Quantitative RT–PCR (qRT-PCR) showed 35.59% increased retroviral activity in sera of SZ patients compared to 2.83% activity in normal individuals. Also, overexpression of syncytin-1 in human U251 glioma cells increases the expression of brain-derived neurotrophic factor (BDNF), neurotrophic tyrosine kinase receptor type (NTRK2), and dopamine receptor D3 (DRD3) which contribute to the pathogenesis of the recent-onset SZ at both the mRNA and the protein levels determined by qRT- PCR and western blot (Lewis and Lieberman 2000; Woo et al. 2020). The luciferase assay reveals that syncytin-1 significantly activates BDNF promoter activity in human U251 glioma cells. Therefore, the data suggest that syncytin-1 increases the promoter activation of BDNF, NTRK2, and DRD3 and increases the expression of these genes in neuroglia cells.

SZ patients and patients with other neurological disorders such as Alzheimer's disease display calcium imbalance (Berridge 2012). Syncytin-1 induces Ca^2+^ influx in human neuroblastoma cells and upregulates the expression of transient receptor potential channel C3 (TRPC3) via directly regulating its expression or downregulating disrupted-in-schizophrenia 1 (DISC1), a susceptibility factor for SZ, which is a scaffold protein interacting with various proteins to regulate synaptic processes and dopamine signaling (Chen et al. 2019; Kim et al. 2020).

### HERVs and COVID-19

COVID-19 patients exhibit increased prevalence of psychological conditions and HERVs are often highly expressed in people with COVID-19 infection. Therefore, HEVRs might contribute to the process of viral penetration and the onset of symptoms in COVID-19 disease.

Based on a questionnaire with 205 participants regarding psychological distress, COVID-19 patients show a predominantly increased prevalence of depression (Zhang et al. 2020). Another study shows that 96.2% out of 714 hospitalized but clinically stable COVID-19 patients suffered from significant post-traumatic stress disorder (PTSD) symptoms before discharge (Bo et al. 2021). Several COVID-19 patients with no previous psychiatric history experience psychotic symptoms characterized by thoughts of reference and structured delusional beliefs as in primary psychosis conditions (Rentero et al. 2020). Some patients also showed acute delirium, and those were the only patients who also suffered from hallucinations. Another case of symptomatic COVID-19-related psychosis in a 36-year-old patient with no personal or family history of mental illness is reported (Smith et al. 2020). The patient shows an acute and rapidly progressive change in behavior characterized by reduced sleep and delusions. Antipsychotics and benzodiazepines helped to improve psychotic symptoms, and further improvement was seen with the resolution of COVID-19 symptoms.

A follow-up study after 6 months of diagnosis of 182 COVID-19 patients show that 91.2% of the patients had psychiatric symptoms, such as poor sleep (64.8%), PTSD (28.6%), somatization (41.8%), obsessive-compulsive (19.8%), depression (11.5%), anxiety (28%), phobic-anxiety (24.2%) and psychoticism (17.6%) (Ahmed et al. 2021). The critical group regarding the severity of COVID-19 infection presents the highest percentages of PTSD, anxiety, and psychosis. The non-severe group had the least CRP level. The study among a total of 27 patients (11 mild cases, 12 moderate cases, 2 severe cases, and 2 critical cases) revealed CRP levels positively correlated with the lung lesion and disease severity (Wang 2020). CRP levels and the diameter of the largest lung lesion increased as the disease progressed.

Another case study also showed that 3 patients who did not show any physical symptoms for COVID-19 but displayed new-onset psychotic symptoms including anxiety, suicidal ideation, agitation, suspiciousness, auditory hallucinations, paranoid and persecutory delusions (Zhang et al. 2019; Ferrando et al. 2020). They were all further tested positive for COVID-19 even though no respiratory or gastrointestinal symptoms, and vital signs, complete blood count, comprehensive metabolic panel, chest x-ray, and brain computed tomography (CT) scan were normal. However, all patients had increased inflammatory markers, notably CRP. CRP involves in triggering schizophreniform psychosis, which is similar to SZ, but usually lasts for less than 6 months (Miller et al. 2014). Thus, there might be a possible virus-associated inflammatory trigger because of elevated CRP level in SARS-CoV-2 infection in these COVID-19 patients.

HERVs may play a role in SARS-CoV-2 infection and COVID-19 symptoms (El-Shehawi et al. 2020). HERVs or their viral products might promote the infection and penetrance of the virus to human cells, or HERVs may produce proteins that regulate the translation start for the ribosome changing the pattern of COVID-19 ORFs in different human hosts; thus the severity and course of the COVID-19 infection would differ.

According to a transcriptome analysis, the expression of HERV-FRD, HERV-H, HERV-W, HERV-L, HERV-I, HERV-K (HML-5), HERV-K (HML-3) and HERV-K (HML-1) is significantly upregulated in bronchoalveolar lavage fluid (BALF) of COVID-19 patients compared to that in BALF of healthy individuals but not in the peripheral blood monocytes (PBMCs) (Kitsou et al. 2021). HERV-FRD is the most highly upregulated family among other HERVs. Another recent study supports the finding of upregulated HERVs in BALF of COVID-19 patients (Marston et al. 2021). Both syncytin-1 and syncytin-2 are highly expressed in BALF of COVID-19 patients, but none are detected in PBMCs (Charvet et al. 2021). SARS-CoV-2 activates syncytin-1 and HERV-K env transcription *in vitro* through the interaction between spike proteins and ACE2 on T lymphocytes.

Syncytin-1 is highly expressed in the leukocytes of 30 hospitalized COVID-19 patients with a wide range of severity of illnesses (Balestrieri et al. 2021; Garcia-Montojo and Nath 2021). The expression of syncytin-1 is correlated with the inflammatory markers and the severity of illness in COVID-19 patients. A study in pediatric COVID-19 patients shows that the expression of HERVs in children with mild symptoms is higher than in children with severe symptoms or with multisystem inflammatory syndrome MIS-C (Tovo et al. 2021). Patients are categorized into four groups; 36 children with mild/moderate symptoms, 17 children with severe symptoms and 11 children with MIS-C. Transcriptional levels of *pol* genes of HERV-H, HERV-K, and HERV-W, and of *env* genes of syncytin-1, syncytin-2, and MSRV are varied among four groups. Children with mild symptoms have the highest expression for HERV-H-*pol,* HERV-K-*pol,* syncytin-1, and syncytin-2. In contrast, children with severe symptoms show significantly lower HERV-W-*pol* and MSRV-*env* than the control group, but a comparable expression of HERV-H-*pol* and HERV-K-*pol* to the control. The correlation between HERVs and SARS-CoV-2-infected children suggests that HERVs might involve in the infection process.

## Infertility concerns: now all over

During the COVID-19 pandemic, there is still high vaccine hesitancy among people. In addition to safety concerns handling and administering vaccines, there is an opinion that it is unnecessary to vaccinate against COVID-19 because the disease is considered not life-threatening. Also, some lack trust on the administration and doubts about the efficiency of vaccination. Above all, some scientists warned that vaccination might cause infertility in women at the early stage of vaccine development (Dror et al. 2020; Troiano and Nardi 2021). Hesitancy regarding vaccination is higher among the younger and female population, who might have been influenced by possible infertility associated with vaccines, which is now turned out to be out of concern. The false concept that COVID-19 vaccines might cause infertility emerged from a claim that antibodies against products of COVID-19 vaccines could attack the placenta since these antibodies of SARS-CoV-2 spike protein can cross-react with the human placental protein syncytin-1, the ENV protein of HERV-W (Male 2021). However, several fertility and obstetric organizations released statements that there is no evidence linking the vaccine to infertility (Schaler and Wingfield 2021).

First of all, the BLAST comparison between the amino acid sequences of human syncytin-1 (538aa) and SARS-CoV-2 spike protein (1273aa) indicates no homology between these two proteins; They are similar in only one 5-amino acid stretches, VVLQN for syncytin-1 and VVNQN for the spike protein, only two 2-amino acids identities (VV and QN). Considering the lack of homology between these two proteins, it is very unlikely that any antibodies raised against SARS-CoV-2 spike protein cross-react with endogenous human syncytin-1 (Kloc et al. 2021). Scientific studies show no cross-reactivity of anti-spike protein antibodies with syncytin-1 (Prasad et al. 2021). In the study, a stable HEK293 cell line expressing syncytin-1 on the cell surface is generated and anti-spike protein antibody activity was tested by an in-cell ELISA. None of the ten monoclonal antibodies tested bind to syncytin-1. Although the amount of anti-spike protein was increased in the plasma of vaccinated individuals, any cross-reactivity was detected with syncytin-1. In addition, no anti-syncytin-1 antibodies were detected in the plasma of COVID-19 patients. These data clearly show that there is no cross-reactivity between antibodies against a SARS-CoV-2 spike protein and endogenous syncytin-1.

Expectedly, a cohort study conducted in 3,958 pregnant women shows no risk of increased miscarriage in women vaccinated in early pregnancy, compared to the general female population (Shimabukuro et al. 2021). Also, a clinical study conducted in three countries of UK, Brazil, and South Africa indicates that the adenovirus-based vector COVID-19 vaccine shows no increased risk of miscarriage and reduced fertility in women who received the vaccine before pregnancy. Among 9755 participants, 121 pregnancies were reported during trials (Hillson et al. 2021). The fertility outcome analysis set included 93 pregnant women, 50 of who received the COVID-19 vaccine, and 43 of whom received the control vaccine. No evidence was found for a relation between reduced fertility and vaccination COVID-19 vaccine. The pregnancy outcome analysis set included 72 of whom received the COVID-19 vaccine, and 35 received the control vaccine. Global clinical trials of COVID-19 vaccine found no increased risk of miscarriage and instances of stillbirth in women vaccinated before pregnancy, compared with women who received the control vaccine (Hillson et al. 2021). Finally, a study using frozen embryo transfer (FET) in women shows no difference in embryo implantation rates or early pregnancy development between SARS-CoV-2 vaccine seropositive, infection seropositive, and seronegative women (Morris 2021). Thus, no evidence is found regarding COVID-19 vaccines resulting in female sterility.

## Conclusion

HERVs have been participating in human evolution. Human development requires HERV ENV proteins to build a highly elaborate placenta. When dysregulated, those proteins unbalance cellular activities to aggravate human diseases such as cancers and mental illnesses. Recent studies indicate that the essential HERV ENV proteins may be involved in developing the psychotic and cognitive symptoms of COVID-19 disease. Based on clear scientific evidence, the speculation regarding COVID-19 vaccinations causing infertility due to a misunderstanding of viral protein structure has been proved wrong. HERV activities in COVID-19 disease strongly suggest that the ancient relics in the human genome play a significant role in human evolution even in the unprecedented pandemic of COVID-19.

## References

[CIT0001] Ahmed GK, Khedr EM, Hamad DA, Meshref TS, Hashem MM, Aly MM. 2021. Long term impact of Covid-19 infection on sleep and mental health: a cross-sectional study. Psychiatry Res. 305:114243. 34673325 10.1016/j.psychres.2021.114243 PMC8507572

[CIT0002] Alcazer V, Bonaventura P, Depil S. 2020. Human endogenous retroviruses (HERVs): shaping the innate immune response in cancers. Cancers (Basel). 12:610. 10.3390/cancers12030610 PMC7139688 32155827

[CIT0003] Antony JM, Van Marle G, Opii W, Butterfield DA, Mallet F, Yong VW, Wallace JL, Deacon RM, Warren K, Power C. 2004. Human endogenous retrovirus glycoprotein-mediated induction of redox reactants causes oligodendrocyte death and demyelination. Nat Neurosci. 7:1088–1095. 15452578 10.1038/nn1319

[CIT0004] Balada E, Ordi-Ros J, Vilardell-Tarrés M. 2009. Molecular mechanisms mediated by human endogenous retroviruses (HERVs) in autoimmunity. Rev Med Virol. 19:273–286. 19714703 10.1002/rmv.622

[CIT0005] Balestrieri E, Minutolo A, Petrone V, Fanelli M, Iannetta M, Malagnino V, Zordan M, Vitale P, Charvet B, Horvat B, et al. 2021. Evidence of the pathogenic HERV-W envelope expression in T lymphocytes in association with the respiratory outcome of COVID-19 patients. EBioMedicine. 66:103341. 33867312 10.1016/j.ebiom.2021.103341 PMC8082064

[CIT0006] Barbulescu M, Turner G, Seaman MI, Deinard AS, Kidd KK, Lenz J. 1999. Many human endogenous retrovirus K (HERV-K) proviruses are unique to humans. Curr Biol. 9:861–868. 10469592 10.1016/s0960-9822(99)80390-x

[CIT0007] Benirschke K, Burton GJ, Baergen RN. 2012. Early development of the human placenta. Pathol Hum Placenta. 1:41–53.

[CIT0008] Berridge MJ. 2012. Calcium signalling remodelling and disease. Biochem Soc Trans. 40:297–309. 22435804 10.1042/BST20110766

[CIT0009] Blaise S, De Parseval N, Bénit L, Heidmann T. 2003. Genomewide screening for fusogenic human endogenous retrovirus envelopes identifies syncytin 2, a gene conserved on primate evolution. Proc Natl Acad Sci. 100:13013–13018. 14557543 10.1073/pnas.2132646100 PMC240736

[CIT0010] Blond J-L, Lavillette D, Cheynet V, Bouton O, Oriol G, Chapel-Fernandes S, Mandrand B, Mallet F, Cosset F-L. 2000. An envelope glycoprotein of the human endogenous retrovirus HERV-W is expressed in the human placenta and fuses cells expressing the type D mammalian retrovirus receptor. J Virol. 74:3321–3329. 10708449 10.1128/jvi.74.7.3321-3329.2000 PMC111833

[CIT0011] Bo H-X, Li W, Yang Y, Wang Y, Zhang Q, Cheung T, Wu X, Xiang Y-T. 2021. Posttraumatic stress symptoms and attitude toward crisis mental health services among clinically stable patients with COVID-19 in China. Psychol Med. 51:1. 32216863 10.1017/S0033291720000999 PMC7200846

[CIT0012] Bokslag A, van Weissenbruch M, Mol BW, de Groot CJM. 2016. Preeclampsia; short and long-term consequences for mother and neonate. Early Hum Dev. 102:47–50. 27659865 10.1016/j.earlhumdev.2016.09.007

[CIT0013] Cavalcante MB, Cavalcante CTdMB, Sarno M, Barini R, Kwak-Kim J. 2021. Maternal immune responses and obstetrical outcomes of pregnant women with COVID-19 and possible health risks of offspring. J Reprod Immunol. 143:103250. 33249335 10.1016/j.jri.2020.103250 PMC7676367

[CIT0014] Chang C, Chen PT, Chang GD, Huang CJ, Chen H. 2004. Functional characterization of the placental fusogenic membrane protein syncytin. Biol Reprod. 71:1956–1962. 15269105 10.1095/biolreprod.104.033340

[CIT0015] Charvet B, Brunel J, Pierquin J, Mathieu C, Perron H. 2021. SARS-CoV-2 induces transcription of human endogenous retrovirus RNA followed by type W envelope protein expression in human lymphoid cells.

[CIT0016] Chen W, Zheng KI, Liu S, Yan Z, Xu C, Qiao Z. 2020. Plasma CRP level is positively associated with the severity of COVID-19. Ann Clin Microbiol Antimicrob. 19:18. 32414383 10.1186/s12941-020-00362-2 PMC7227180

[CIT0017] Chen Y, Yan Q, Zhou P, Li S, Zhu F. 2019. HERV-W env regulates calcium influx via activating TRPC3 channel together with depressing DISC1 in human neuroblastoma cells. J NeuroVirology. 25:101–113. 30397826 10.1007/s13365-018-0692-7

[CIT0018] Chignola R, Sega M, Molesini B, Baruzzi A, Stella S, Milotti E, Ahmad A. 2019. Collective radioresistance of T47D breast carcinoma cells is mediated by a syncytin-1 homologous protein. PLoS One. 14:e0206713. 30699112 10.1371/journal.pone.0206713 PMC6353071

[CIT0019] Cianciolo GJ, Copeland TD, Oroszlan S, Snyderman R. 1985. Inhibition of lymphocyte proliferation by a synthetic peptide homologous to retroviral envelope proteins. New Ser. 230:453–455. 10.1126/science.2996136 2996136

[CIT0020] Dolei A, Ibba G, Piu C, Serra C. 2019. Expression of HERV genes as possible biomarker and target in neurodegenerative diseases. Int J Mol Sci. 20:3706. 10.3390/ijms20153706 PMC6696274 31362360

[CIT0021] Dror AA, Eisenbach N, Taiber S, Morozov NG, Mizrachi M, Zigron A, Srouji S, Sela E. 2020. Vaccine hesitancy: the next challenge in the fight against COVID-19. Eur J Epidemiol. 35:775–779. 32785815 10.1007/s10654-020-00671-y PMC8851308

[CIT0022] Du Clos TW, Mold C. 2004. C-Reactive protein: an activator of Innate immunity and a modulator of adaptive immunity. Immunol Res. 30:261–278. 15531769 10.1385/IR:30:3:261

[CIT0023] Dupressoir A, Lavialle C, Heidmann T. 2012. From ancestral infectious retroviruses to bona fide cellular genes: role of the captured syncytins in placentation. Placenta. 33:663–671. 22695103 10.1016/j.placenta.2012.05.005

[CIT1001] Durnaoglu S, Lee S-K, Ahnn J. 2021. Human endogenous retroviruses as gene expression regulators: insights from animal models into human diseases. Mol Cells. 44:861–878.

[CIT0024] El-Shehawi AM, Alotaibi SS, Elseehy MM. 2020. Genomic study of COVID-19 corona virus excludes its origin from recombination or characterized biological sources and suggests a role for HERVS in its wide range symptoms. Cytol Genet. 54:588–604. 33487779 10.3103/S0095452720060031 PMC7810191

[CIT0025] Ferrando SJ, Klepacz L, Lynch S, Tavakkoli M, Dornbush R, Baharani R, Smolin Y, Bartell A. 2020. COVID-19 psychosis: a potential new neuropsychiatric condition triggered by novel coronavirus infection and the inflammatory response? Psychosomatics. 61:551–555. 32593479 10.1016/j.psym.2020.05.012 PMC7236749

[CIT0026] Frendo J-L, Olivier D, Cheynet V, Blond J-L, Bouton O, Vidaud M, Rabreau M, Evain-Brion D, Mallet F. 2003. Direct involvement of HERV-W Env glycoprotein in human trophoblast cell fusion and differentiation. Mol Cell Biol. 23:3566–3574. 12724415 10.1128/MCB.23.10.3566-3574.2003 PMC164757

[CIT0027] Gao Y, Yu X-F, Chen T. 2021. Human endogenous retroviruses in cancer: expression, regulation and function. Oncol Lett. 21:121–132. 33552242 10.3892/ol.2020.12382 PMC7798031

[CIT0028] Garcia-Montojo M, Nath A. 2021. HERV-W envelope expression in blood leukocytes as a marker of disease severity of COVID-19. EBioMedicine. 67:103363. 33993053 10.1016/j.ebiom.2021.103363 PMC8116818

[CIT0029] Garcia-Montojo M, Rodriguez-Martin E, Ramos-Mozo P, Ortega-Madueño I, Dominguez-Mozo MI, Arias-Leal A, García-Martínez MÁ, Casanova I, Galan V, Arroyo R, et al. 2020. Syncytin-1/HERV-W envelope is an early activation marker of leukocytes and is upregulated in multiple sclerosis patients. Eur J Immunol. 50:685–694. 32012247 10.1002/eji.201948423

[CIT0030] Gimenez J, Mallet F. 2008. Gene section Atlas of genetics and cytogenetics in oncology and haematology Open access journal at INIST-CNRS ERVWE1 (endogenous retroviral family W, Env(C7), member 1). Atlas Genet Cytogenet Oncol Haematol. 12:134–148.

[CIT0031] Gong R, Peng X, Kang S, Feng H, Huang J, Zhang W, Lin D, Tien P, Xiao G. 2005. Structural characterization of the fusion core in syncytin, envelope protein of human endogenous retrovirus family W. Biochem Biophys Res Commun. 331:1193–1200. 15883002 10.1016/j.bbrc.2005.04.032 PMC7092852

[CIT0032] Grandi N, Tramontano E. 2018. HERV envelope proteins: physiological role and pathogenic potential in cancer and autoimmunity. Front Microbiol. 9:462. 29593697 10.3389/fmicb.2018.00462 PMC5861771

[CIT0033] Griffiths DJ. 2001. Endogenous retroviruses in the human genome sequence. Genome Biol. 2:1017.1–1017.5. 10.1186/gb-2001-2-6-reviews1017 PMC138943 11423012

[CIT0034] Hillson K, Clemens SC, Madhi SA, Voysey M, Pollard AJ, Minassian AM, Group OCVT. 2021. Fertility rates and birth outcomes after ChAdOx1 nCoV-19 (AZD1222) vaccination. Lancet (London, England). 398(10312):1683–1684. 10.1016/S0140-6736(21)02282-0 PMC8530465 34688353

[CIT0035] Huang Q, Chen H, Li J, Oliver M, Ma X, Byck D, Gao Y, Jiang SW. 2014. Epigenetic and non-epigenetic regulation of syncytin-1 expression in human placenta and cancer tissues. Cell Signal. 26:648–656. 24216608 10.1016/j.cellsig.2013.11.002

[CIT0036] Huang W, Li S, Hu Y, Yu H, Luo F, Zhang Q, Zhu F. 2011. Implication of the env gene of the human endogenous retrovirus W family in the expression of BDNF and DRD3 and development of recent-onset schizophrenia. Schizophr Bull. 37:988–1000. 20100784 10.1093/schbul/sbp166 PMC3160218

[CIT0037] Ito J, Sugimoto R, Nakaoka H, Yamada S, Kimura T, Hayano T, Inoue I, Feschotte C. 2017. Systematic identification and characterization of regulatory elements derived from human endogenous retroviruses. PLOS Genet. 13:e1006883. 28700586 10.1371/journal.pgen.1006883 PMC5529029

[CIT0038] Jing Y, Run-Qian L, Hao-Ran W, Hao-Ran C, Ya-Bin L, Yang G, Fei C. 2020. Potential influence of COVID-19/ACE2 on the female reproductive system. Mol Hum Reprod. 26:367–373. 32365180 10.1093/molehr/gaaa030 PMC7239105

[CIT0039] Karlsson H, Bachmann S, Schröder J, McArthur J, Torrey EF, Yolken RH. 2001. From the cover: retroviral RNA identified in the cerebrospinal fluids and brainsof individuals with schizophrenia. Proc Natl Acad Sci. 98:4634–4639. 11296294 10.1073/pnas.061021998 PMC31886

[CIT0040] Khodosevich K, Lebedev Y, Sverdlov E. 2002. Endogenous retroviruses and human evolution. Comp Funct Genomics. 3:494–498. 18629260 10.1002/cfg.216 PMC2448423

[CIT0041] Kim J, Shin C-G. 2020. IFITM proteins inhibit the late step of feline foamy virus replication. Animal Cells Syst (Seoul) [Internet]. 24:282–288. Available from: doi:10.1080/19768354.2020.1819413. PMC7646556 33209202

[CIT0042] Kim YH, Lee S, Yang H, Chun YL, Kim D, Yeo SG, Park C, Jung J, Huh Y. 2020. Inhibition of transient receptor potential melastatin 7 (TRPM7) protects against Schwann cell trans-dedifferentiation and proliferation during Wallerian degeneration. Animal Cells Syst (Seoul) [Internet]. 24:189–196. Available from: doi:10.1080/19768354.2020.1804445. PMC7473164 33029295

[CIT0043] Kitsou K, Kotanidou A, Paraskevis D, Karamitros T, Katzourakis A, Tedder R, Hurst T, Sapounas S, Kotsinas A, Gorgoulis V, et al. 2021. Upregulation of Human Endogenous Retroviruses in Bronchoalveolar Lavage Fluid of COVID-19 Patients. Sinclair A, editor. Microbiol Spectr. 9. 10.1128/Spectrum.01260-21 PMC8510252 34612698

[CIT0044] Kloc M, Uosef A, Kubiak JZ, Ghobrial RM. 2021. Exaptation of retroviral syncytin for development of syncytialized placenta, its limited homology to the SARS-CoV-2 spike protein and arguments against disturbing narrative in the context of COVID-19 vaccination. Biology (Basel). 10:238–250. 33808658 10.3390/biology10030238 PMC8003504

[CIT0045] Kristensen MK, Christensen T. 2021. Regulation of the expression of human endogenous retroviruses: elements in fetal development and a possible role in the development of cancer and neurological diseases. APMIS. 129:241–253. 33683784 10.1111/apm.13130

[CIT0046] Lander ES, Linton LM, Birren B, Nusbaum C, Zody MC, Baldwin J, Devon K, Dewar K, Doyle M, Fitzhugh W, et al. 2001. Initial sequencing and analysis of the human genome. Nature. 409:860–921. 11237011 10.1038/35057062

[CIT0047] Lavillette D, Marin M, Ruggieri A, Mallet F, Cosset F-L, Kabat D. 2002. The envelope glycoprotein of human endogenous retrovirus type W uses a divergent family of amino acid transporters/cell surface receptors. J Virol. 76:6442–6452. 12050356 10.1128/JVI.76.13.6442-6452.2002 PMC136247

[CIT0048] Lewis DA, Lieberman JA. 2000. Catching up on schizophrenia: natural history and neurobiology. Neuron. 28:325–334. 11144342 10.1016/s0896-6273(00)00111-2

[CIT0049] Liu C, Xu J, Wen F, Yang F, Li X, Geng D, Li L, Chen J, Zheng J. 2018. Upregulation of syncytin-1 promotes invasion and metastasis by activating epithelial-mesenchymal transition-related pathway in endometrial carcinoma. Onco Targets Ther. 12:31–40. 30588028 10.2147/OTT.S191041 PMC6301305

[CIT0050] Lokossou AG, Toudic C, Barbeau B. 2014. Implication of human endogenous retrovirus envelope proteins in placental functions. Viruses. 6:4609–4627. 25421890 10.3390/v6114609 PMC4246240

[CIT0051] Lokossou AG, Toudic C, Nguyen PT, Elisseeff X, Vargas A, Rassart É, Lafond J, Leduc L, Bourgault S, Gilbert C, et al. 2020. Endogenous retrovirus-encoded syncytin-2 contributes to exosome-mediated immunosuppression of T cells†. Biol Reprod. 102:185–198. 31318021 10.1093/biolre/ioz124

[CIT0052] Lorkiewicz P, Waszkiewicz N. 2021. Biomarkers of post-COVID depression. J Clin Med. 10:4142. 34575258 10.3390/jcm10184142 PMC8470902

[CIT0053] Łoś K, Kulikowska J, Waszkiewicz N. 2021. First-time psychotic symptoms in a patient after COVID-19 infection—a case report. Front Psychiatry. 12:726059–726059. 34721104 10.3389/fpsyt.2021.726059 PMC8554044

[CIT0054] Lovato A, de Filippis C, Marioni G. 2020. Upper airway symptoms in coronavirus disease 2019 (COVID-19). Am J Otolaryngol. 41:102474–102475. 32278470 10.1016/j.amjoto.2020.102474 PMC7128936

[CIT0055] Lu Q, Li J, Senkowski C, Tang Z, Wang J, Huang T, Wang X, Terry K, Brower S, Glasgow W, et al. 2015. Promoter hypermethylation and decreased expression of syncytin-1 in pancreatic adenocarcinomas. PLoS One. 10:e0134412. 26230721 10.1371/journal.pone.0134412 PMC4521816

[CIT0056] Luo X, Zhou W, Yan X, Guo T, Wang B, Xia H, Ye L, Xiong J, Jiang Z, Liu Y, et al. 2020. Prognostic value of C-reactive protein in patients with COVID-19. Clin Infect Dis. 71:2174–2179. 32445579 10.1093/cid/ciaa641 PMC7314209

[CIT0057] Male V. 2021. Are COVID-19 vaccines safe in pregnancy? Nat Rev Immunol. 21:1. 33658707 10.1038/s41577-021-00525-y PMC7927763

[CIT0058] Marchler-Bauer A, Bo Y, Han L, He J, Lanczycki CJ, Lu S, Chitsaz F, Derbyshire MK, Geer RC, Gonzales NR, et al. 2017. CDD/SPARCLE: functional classification of proteins via subfamily domain architectures. Nucleic Acids Res. 45:D200–D203. 27899674 10.1093/nar/gkw1129 PMC5210587

[CIT0059] Marston JL, Greenig M, Singh M, Bendall ML, Duarte RRR, Feschotte C, Iñiguez LP, Nixon DF. 2021. SARS-CoV-2 infection mediates differential expression of human endogenous retroviruses and long interspersed nuclear elements. JCI Insight. 6(24):e147170. 34731091 10.1172/jci.insight.147170 PMC8783694

[CIT0060] Miller BJ, Culpepper N, Rapaport MH. 2014. C-reactive protein levels in schizophrenia: a review and meta-analysis. Clin Schizophr Relat Psychoses. 7:223–230. 23428789

[CIT0061] Mi S, Lee X, Li X, Veldman GM, Finnerty H, Racie L, LaVallie E, Tang XY, Edouard P, Howes S, et al. 2000. Syncytin is a captive retroviral envelope protein involved in human placental morphogenesis. Nat. 403:785–789. 10.1038/35001608 10693809

[CIT0062] Morris RS. 2021. SARS-CoV-2 spike protein seropositivity from vaccination or infection does not cause sterility. F S Rep. 2:253–255. 34095871 10.1016/j.xfre.2021.05.010 PMC8169568

[CIT0063] Mosquera-Sulbaran JA, Pedreañez A, Carrero Y, Callejas D. 2021. C-reactive protein as an effector molecule in Covid-19 pathogenesis. Rev Med Virol. 31:e2221. 34773448 10.1002/rmv.2221 PMC7995022

[CIT0064] Nehring SM, Goyal A, Bansal P, Patel BC. 2021. C reactive protein. StatPearls. 65:237–244.

[CIT0065] Nguyen LN, Ma D, Shui G, Wong P, Cazenave-Gassiot A, Zhang X, Wenk MR, Goh ELK, Silver DL. 2014. Mfsd2a is a transporter for the essential omega-3 fatty acid docosahexaenoic acid. Nat. 509:503–506. 10.1038/nature13241 24828044

[CIT0066] Okae H, Toh H, Sato T, Hiura H, Takahashi S, Shirane K, Kabayama Y, Suyama M, Sasaki H, Arima T. 2018. Derivation of human trophoblast stem cells. Cell Stem Cell. 22:50–63.e6. 29249463 10.1016/j.stem.2017.11.004

[CIT0067] Osimo EF, Baxter L, Stochl J, Perry BI, Metcalf SA, Kunutsor SK, Laukkanen JA, Wium-Andersen MK, Jones PB, Khandaker GM. 2021. Longitudinal association between CRP levels and risk of psychosis: a meta-analysis of population-based cohort studies. NPJ Schizophr. 7:31–39. 34050185 10.1038/s41537-021-00161-4 PMC8163886

[CIT0068] Park C, HwangBo H, Lee H, Kim G-Y, Cha H-J, Choi SH, Kim S, Kim H-S, Yun SJ, Kim W-J, et al. 2020. The immunostimulatory effect of indole-6-carboxaldehyde isolated from Sargassum thunbergii (Mertens) Kuntze in RAW 264.7 macrophages. Animal Cells Syst (Seoul) [Internet]. 24:233–241. Available from: doi:10.1080/19768354.2020.1808529. PMC7473310 33029301

[CIT0069] Perron H, Hamdani N, Faucard R, Lajnef M, Jamain S, Daban-Huard C, Sarrazin S, LeGuen E, Houenou J, Delavest M, et al. 2012. Molecular characteristics of human endogenous retrovirus type-W in schizophrenia and bipolar disorder. Transl Psychiatry. 2:e201. 23212585 10.1038/tp.2012.125 PMC3565190

[CIT0070] Perron H, Mekaoui L, Bernard C, Veas F, Stefas I, Leboyer M. 2008. Endogenous retrovirus type w GAG and envelope protein antigenemia in serum of schizophrenic patients. Biol Psychiatry. 64:1019–1023. 18760403 10.1016/j.biopsych.2008.06.028

[CIT0071] Petropoulos S, Edsgärd D, Reinius B, Deng Q, Panula SP, Codeluppi S, Plaza Reyes A, Linnarsson S, Sandberg R, Lanner F. 2016. Single-Cell RNA-Seq reveals lineage and X chromosome dynamics in human preimplantation embryos. Cell. 165:1012–1026. 27062923 10.1016/j.cell.2016.03.023 PMC4868821

[CIT0072] Prasad M, Lin JL, Gu Y, Gupta R, Macary P, Schwarz H. 2021. No crossreactivity of anti-SARS-CoV-2 spike protein antibodies with syncytin-1. Cell Mol Immunol. 18:1. 34645941 10.1038/s41423-021-00773-x PMC8513556

[CIT0073] Rajak P, Roy S, Dutta M, Podder S, Sarkar S, Ganguly A, Mandi M, Khatun S. 2021. Understanding the cross-talk between mediators of infertility and COVID-19. Reprod Biol [Internet]. 21:100559. Available from: https://www.sciencedirect.com/science/article/pii/S1642431X21000802. 10.1016/j.repbio.2021.100559 PMC8407955 34547545

[CIT0074] Rentero D, Juanes A, Losada CP, Álvarez S, Parra A, Santana V, Martí I, Urricelqui J. 2020. New-onset psychosis in COVID-19 pandemic: a case series in Madrid. Psychiatry Res. 290:113097. 32480119 10.1016/j.psychres.2020.113097 PMC7217785

[CIT0075] Roberts RM, Ezashi T, Schulz LC, Sugimoto J, Schust DJ, Khan T, Zhou J. 2021. Syncytins expressed in human placental trophoblast. Placenta. 113:8–14. 33504453 10.1016/j.placenta.2021.01.006 PMC8280254

[CIT0076] Rolland A, Jouvin-Marche E, Viret C, Faure M, Perron H, Marche PN. 2006. The Envelope protein of a human endogenous retrovirus-W family activates innate immunity through CD14/TLR4 and promotes Th1-Like responses. J Immunol. 176:7636–7644. 16751411 10.4049/jimmunol.176.12.7636

[CIT0077] Rowe HM, Trono D. 2011. Dynamic control of endogenous retroviruses during development. Virology. 411:273–287. 21251689 10.1016/j.virol.2010.12.007

[CIT0078] Schaler L, Wingfield M. 2021. COVID-19 vaccine — can it affect fertility? Ir J Med Sci. 1:1–3. 10.1007/s11845-021-02807-9 PMC8516490 34651258

[CIT0079] Sharma I, Kumari P, Sharma A, Saha SC. 2021. SARS-CoV-2 and the reproductive system: known and the unknown..!!. Middle East Fertil Soc J. 26:1–9. 33437145 10.1186/s43043-020-00046-z PMC7789900

[CIT0080] Shen Q, Xiao X, Aierken A, Yue W, Wu X, Liao M, Hua J. 2020. The ACE2 expression in sertoli cells and germ cells may cause male reproductive disorder after SARS-CoV-2 infection. J Cell Mol Med. 24:9472–9477. 32594644 10.1111/jcmm.15541 PMC7361928

[CIT0081] Shimabukuro TT, Kim SY, Myers TR, Moro PL, Oduyebo T, Panagiotakopoulos L, Marquez PL, Olson CK, Liu R, Chang KT, et al. 2021. Preliminary findings of mRNA Covid-19 vaccine safety in pregnant persons. N Engl J Med. 384:2273–2282. 33882218 10.1056/NEJMoa2104983 PMC8117969

[CIT0082] Smith CM, Komisar JR, Mourad A, Kincaid BR. 2020. Case report: COVID-19-associated brief psychotic disorder. BMJ Case Rep. 13:e236940. 10.1136/bcr-2020-236940 PMC7418683 32784244

[CIT0083] Sun Y, Zhu H, Song J, Jiang Y, Ouyang H, Dong T, Tao R, Fan X, Zhang G. 2017. Expression of leukocytic syncytin-1 in B-cell acute lymphoblastic leukemia and acute myeloid leukemia patients. Clin Lab. 63:1567–1574. 29035445 10.7754/Clin.Lab.2017.170116

[CIT0084] Tolosa JM, Schjenken JE, Clifton VL, Vargas A, Barbeau B, Lowry P, Maiti K, Smith R. 2012. The endogenous retroviral envelope protein syncytin-1 inhibits LPS/PHA-stimulated cytokine responses in human blood and is sorted into placental exosomes. Placenta. 33:933–941. 22999499 10.1016/j.placenta.2012.08.004

[CIT0085] Tovo P-A, Garazzino S, Daprà V, Pruccoli G, Calvi C, Mignone F, Alliaudi C, Denina M, Scolfaro C, Zoppo M, et al. 2021. COVID-19 in children: expressions of type I/II/III interferons, TRIM28, SETDB1, and endogenous retroviruses in mild and severe cases. Int J Mol Sci. 22:7481–7508. 34299101 10.3390/ijms22147481 PMC8303145

[CIT0086] Troiano G, Nardi A. 2021. Vaccine hesitancy in the era of COVID-19. Public Health. 194:245–251. 33965796 10.1016/j.puhe.2021.02.025 PMC7931735

[CIT0087] Vargas A, Toufaily C, Lebellego F, Rassart É, Lafond J, Barbeau B. 2011. Reduced expression of both syncytin 1 and syncytin 2 correlates with severity of preeclampsia. Reprod Sci. 18:1085–1091. 21493955 10.1177/1933719111404608

[CIT0088] Vizheh M, Allahdadian M, Muhidin S, Valiani M, Bagheri K, Borandegi F, Ghasimi G. 2021. Impact of COVID-19 infection on neonatal birth outcomes. J Trop Pediatr. 67:1–8. 10.1093/tropej/fmab094 34748020

[CIT0089] Wang J, Youkharibache P, Zhang D, Lanczycki CJ, Geer RC, Madej T, Phan L, Ward M, Lu S, Marchler GH, et al. 2020. Icn3d, a web-based 3D viewer for sharing 1D/2D/3D representations of biomolecular structures. Bioinformatics. 36:131–135. 31218344 10.1093/bioinformatics/btz502 PMC6956771

[CIT0090] Wang L. 2020. C-reactive protein levels in the early stage of COVID-19. Med Mal Infect. 50:332–334. 32243911 10.1016/j.medmal.2020.03.007 PMC7146693

[CIT0091] Wang X, Liu Z, Wang P, Li S, Zeng J, Tu X, Yan Q, Xiao Z, Pan M, Zhu F. 2018. Syncytin-1, an endogenous retroviral protein, triggers the activation of CRP via TLR3 signal cascade in glial cells. Brain Behav Immun. 67:324–334. 28928004 10.1016/j.bbi.2017.09.009

[CIT0092] West RC, Ming H, Logsdon DM, Sun J, Rajput SK, Kile RA, Schoolcraft WB, Roberts RM, Krisher RL, Jiang Z, Yuan Y. 2019. Dynamics of trophoblast differentiation in peri-implantation–stage human embryos. Proc Natl Acad Sci. 116:22635–22644. 31636193 10.1073/pnas.1911362116 PMC6842583

[CIT0093] Woo DH, Hur Y-N, Jang MW, Justin Lee C, Park M. 2020. Inhibitors of synaptic vesicle exocytosis reduce surface expression of postsynaptic glutamate receptors. Animal Cells Syst (Seoul) [Internet]. 24:341–348. Available from: doi:10.1080/19768354.2020.1838607. PMC7781898 33456718

[CIT0094] Xiao R, Li S, Cao Q, Wang X, Yan Q, Tu X, Zhu Y, Zhu F. 2017. Human endogenous retrovirus W env increases nitric oxide production and enhances the migration ability of microglia by regulating the expression of inducible nitric oxide synthase. Virol Sin. 32:216–225. 28656540 10.1007/s12250-017-3997-4 PMC6598877

[CIT0095] Yabe S, Alexenko AP, Amita M, Yang Y, Schust DJ, Sadovsky Y, Ezashi T, Roberts RM. 2016. Comparison of syncytiotrophoblast generated from human embryonic stem cells and from term placentas. Proc Natl Acad Sci. 113:E2598–E2607. 27051068 10.1073/pnas.1601630113 PMC4868474

[CIT0096] Yu H, Liu T, Zhao Z, Chen Y, Zeng J, Liu S, Zhu F. 2013. Mutations in 3′-long terminal repeat of HERV-W family in chromosome 7 upregulate syncytin-1 expression in urothelial cell carcinoma of the bladder through interacting with c-Myb. Oncogene. 33:3947–3958. 24013223 10.1038/onc.2013.366

[CIT0097] Yuan B, Li W, Liu H, Cai X, Song S, Zhao J, Hu X, Li Z, Chen Y, Zhang K, et al. 2020. Correlation between immune response and self-reported depression during convalescence from COVID-19. Brain Behav Immun. 88:39–43. 32464158 10.1016/j.bbi.2020.05.062 PMC7247486

[CIT0098] Zhang J, Lu H, Zeng H, Zhang S, Du Q, Jiang T, Du B. 2020. The differential psychological distress of populations affected by the COVID-19 pandemic. Brain Behav Immun. 87:49–50. 32304883 10.1016/j.bbi.2020.04.031 PMC7156946

[CIT0099] Zhang Y, Kang HR, Han K. 2019. Differential cell-type-expression of CYFIP1 and CYFIP2 in the adult mouse hippocampus. Animal Cells Syst (Seoul) [Internet]; 23:380–383. [accessed 2020 Mar 6]. Available from: http://www.ncbi.nlm.nih.gov/pubmed/31853374. 10.1080/19768354.2019.1696406 PMC6913624 31853374

[CIT0100] Zhou Y, Liu L, Liu Y, Zhou P, Yan Q, Yu H, Chen X, Zhu F. 2021. Implication of human endogenous retrovirus W family envelope in hepatocellular carcinoma promotes MEK/ERK-mediated metastatic invasiveness and doxorubicin resistance. Cell Death Discov. 7:1–14. 10.1038/s41420-021-00562-5 PMC8266889 34238921

